# Sublingual Crushed Levodopa for Parkinson’s Disease Management of a Lewy Body Dementia Patient With Dysphagia: A Case Report

**DOI:** 10.7759/cureus.85064

**Published:** 2025-05-29

**Authors:** Kristin Regehr, Jessica Yu, Fiona Wu, Reema Shah, Anthony Lau

**Affiliations:** 1 Pharmacy, Vancouver General Hospital, Vancouver, CAN; 2 Hospital Medicine, Vancouver General Hospital, Vancouver, CAN; 3 Emergency Department, Vancouver General Hospital, Vancouver, CAN

**Keywords:** carbidopa, case report, dysphagia, levodopa, parkinson's, sublingual

## Abstract

Levodopa/carbidopa is the cornerstone treatment for Parkinson’s disease (PD), but in patients with dysphagia, there are limited options for the routes of administration. Traditional methods of administering levodopa/carbidopa in these patients, such as rectal administration, have been shown to offer limited symptomatic relief and suboptimal pharmacokinetic outcomes. This case explores sublingual administration of levodopa/carbidopa as a novel solution for managing parkinsonian symptoms in dysphagic patients in the hospital setting, where non-oral options are often limited. We present an 85-year-old Caucasian male with Lewy body dementia and parkinsonian symptoms, who presented to the emergency department with dysphagia and altered level of consciousness in the context of a urinary tract infection. Due to a missed dose of levodopa/carbidopa, he exhibited worsening bradykinesia, rigidity, resting tremors, and dysphagia. Levodopa/carbidopa was crushed and administered sublingually. Symptom improvement and resolution were observed within two hours of the sublingual dose, and the patient was able to tolerate oral medications thereafter. This case demonstrates that sublingual administration of levodopa/carbidopa may be a practical and effective alternative approach for managing parkinsonian symptoms in patients with dysphagia.

## Introduction

Levodopa/carbidopa is the first-line treatment for managing Parkinson’s disease (PD) motor symptoms. Parkinsonian symptoms are a result of decreased dopamine production and availability, where dopamine is the main neurotransmitter responsible for motor function and coordination. Lewy body dementia is an umbrella term used for neurodegenerative disorders that are characterized by alpha-synuclein protein deposits throughout the neocortex, leading to changes in motor function, cognition, and behavior [1]. Levodopa, a dopamine precursor, crosses the blood-brain barrier but is rapidly converted to dopamine in the periphery, limiting its central availability and contributing to its short half-life of 50 minutes [2]. Carbidopa, an aromatic amino acid decarboxylase inhibitor, prevents this peripheral conversion, allowing more levodopa to remain in circulation for transport to the brain. This prolongs levodopa’s duration of action, improves its therapeutic effects, and reduces gastrointestinal side effects and the risk of cardiac arrhythmias [2].

Up to 80% of patients with PD or parkinsonian symptoms will encounter dysphagia at some point in the course of their disease, leading to challenges with medication administration [3]. A common approach to providing levodopa/carbidopa doses to dysphagic PD patients is to administer doses rectally. Studies on rectal levodopa/carbidopa administration suggest limited effectiveness in managing PD symptoms. A 1981 study found no clinical improvement or detectable rise in serum levodopa levels two hours post-dose, along with a lack of improvement in parkinsonian symptoms in any patients who received rectally administered levodopa/carbidopa [4]. Another pharmacokinetic study showed very low concentrations of rectally administered levodopa in comparison to oral administration levodopa [5]. The serum levodopa concentration detected was 17 nmol/L drawn one hour post-dose, when oral levodopa concentrations are often described as 1400-12000 nmol/L. Rectal administration is further complicated by erratic absorption, contributing to the inconsistent and often inadequate therapeutic response. Some factors that contribute to the erratic rectal absorption of drugs include limited contact surface area for absorption and interaction of the drug with fecal matter and rectal mucus, which can disrupt predictable pharmacokinetics and may result in non-linear or mixed-order kinetics [6]. Case reports regarding efficacy note only partial symptom relief along with the aforementioned subtherapeutic levodopa levels, resulting in suboptimal management and increased daily dose requirements. [5,7]

Alternative routes of administration for levodopa/carbidopa in patients with PD and dysphagia include orally disintegrating tablets, subcutaneous foslevodopa/foscarbidopa, and enteral levodopa/carbidopa gel infusion [8]. These options are often unavailable or costly in hospitals, leaving clinicians with limited alternatives for dopamine replacement when oral administration is not feasible or safe. Although levodopa is a hydrophilic molecule with typically low sublingual permeability, some absorption may still occur via passive diffusion or facilitated transport in the highly vascularized sublingual mucosa [9]. This route bypasses first-pass metabolism and may offer faster onset compared to oral administration, but with variable bioavailability. This case report explores sublingual administration as a potential alternative, supported by one previous study, particularly given the lack of formulary options and the inconsistent efficacy of rectal administration [10].

## Case presentation

We present the case of an 85-year-old Caucasian male from a long-term care facility with Lewy body dementia with parkinsonism who presented to the emergency department with confusion and an altered level of consciousness. He was disoriented to place and time, which was different from his baseline, in the context of an acute urinary tract infection and acute kidney injury. He had no recent visits to the emergency department or hospital. Prior to his presentation, he had an overall cognitive decline due to his Lewy body dementia, but his parkinsonian symptoms were well controlled, with decreasing frequency of falls noted at the long-term care facility. His medical history included basal cell carcinoma, benign prostatic hyperplasia, cataracts, cavernous malformation, chronic obstructive pulmonary disease, diverticulosis, a history of left hip fracture with replacement, and Lewy body dementia. His home medications included donepezil 10 mg orally daily, levodopa/carbidopa immediate-release 100 mg/25 mg one tablet orally three times daily, sennosides 24 mg orally nightly, and tiotropium 5 mcg inhaled daily. His vital signs were stable with a temperature of 36.4°C, heart rate of 59 bpm, blood pressure of 139/61 mmHg, respiratory rate of 18 breaths/min, and oxygen saturation of 94% on room air (Table 1). A computed tomography scan of his head was unremarkable. Urinalysis revealed a large presence of leukocytes, and urine culture showed mixed bacterial growth, suggestive of possible contamination. Blood cultures were negative. In the emergency department, the patient was given a dose of IV ceftriaxone 2 g and 1 liter of IV normal saline for the management of his urinary tract infection and rehydration.

**Table 1:** Laboratory results on admission

Parameter	Results on admission	Normal range
White blood cells (x10^9/L)	9.0	4-11
Neutrophils (x10^9/L)	7.0	2-8
Hemoglobin (g/L)	127	135-170
Sodium (mmol/L)	145	135-145
Potassium (mmol/L)	3.8	3.5-5
Serum creatinine (umol/L)	145	55-109
Estimated glomerular filtration rate (mL/min)	37	Greater than 59
Urine leukocytes	Large	Negative

At presentation, the patient had missed his levodopa/carbidopa by about four hours and exhibited notable resting tremors in both hands (approximately two cycles per second), body rigidity, and difficulty swallowing. These symptoms were consistent with a parkinsonian "off" period. He had been prescribed immediate-release levodopa/carbidopa 100/25 mg three times daily for parkinsonian tremors, providing the modest benefit. Due to swallowing difficulties at home, his family had been crushing his medication using a standard handheld pill crusher, resulting in a fine powder that was given orally. Rectal administration was considered but not attempted, as this route of administration had been previously trialed and was found ineffective. The family also expressed concern that rectal administration might cause embarrassment or distress for the patient, who had previously voiced concerns regarding modesty during personal care. On the day of admission, his altered level of consciousness prevented the safe administration of all oral medications at home. 

In the emergency department, he was still unable to take oral medications, and no formulary alternatives for dopaminergic agents were available via another route. We opted to administer levodopa/carbidopa sublingually by crushing the immediate-release tablet and placing it under his tongue. Although this route is not well-established, the sublingual administration was attempted, given the clinical urgency and lack of alternatives. Within two hours of the sublingual dose, his hand tremors improved from a frequency of two cycles per second to barely perceptible, his upper limb rigidity lessened, and he was able to voluntarily lift his right arm above chest level, an action not observed on arrival. These findings were subjectively documented by the treating nurse, as no formal motor scoring was performed. This improvement was clinically consistent with a return to his motor baseline, confirmed by the family members. Within 24 hours of receiving the initial antibiotic dose for treatment of the urinary tract infection, he regained the ability to follow commands and speak with his family, consistent with a return to his neurological baseline.

**Figure 1:**
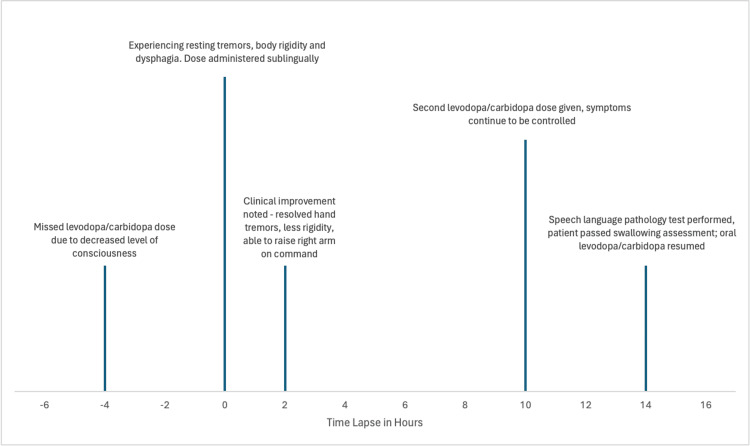
Timeline of events during the patient's hospitalization

Following this improvement, he passed a swallowing assessment, allowing him to resume a dysphagia diet and oral levodopa/carbidopa. Over the next 48 hours, he remained tremor-free, cognitively intact, and actively engaged with staff and family. Please refer to Figure 1 for the chronology of events.

## Discussion

To our knowledge, this is the first case report describing the successful and timely use of sublingual levodopa/carbidopa for the treatment of active parkinsonian symptoms. Although the sublingual route demonstrated apparent clinical benefit in this case, the pharmacokinetics of levodopa/carbidopa via this route are not well established, and the extent, reliability, and consistency of absorption remain uncertain in the absence of serum levodopa concentration monitoring. 

In palliative care settings, where intravenous, oral, or subcutaneous access may be limited, clinicians often rely on alternative routes such as rectal or sublingual/buccal administration. As rectal administration was discouraged by family preference, we extrapolated to this case by selecting the sublingual route as a practical alternative. The dose was kept equivalent to the patient’s usual oral regimen due to the absence of established pharmacokinetic data for sublingual levodopa/carbidopa.

The sublingual route is often an under-recognized and underutilized method of drug administration that allows for rapid absorption directly into the systemic circulation through blood vessels under the tongue, bypassing first-pass metabolism. This results in a faster onset of action with no expected effect on half-life or duration of effect. [11] Furthermore, levodopa absorption is influenced by factors such as delayed gastric emptying, gastric pH, liver metabolism, and concurrent high-protein intake, which could be largely mitigated if given via sublingual administration. [12] Ultimately, pharmacokinetic data for this route remains uncertain, and further research is needed to establish optimal dosing strategies.

Levodopa possesses a small molecular size (~197 Da) and relatively high water solubility (~1.6 g/L), which are typically favorable properties for sublingual administration [13,14]. However, its poor lipophilicity (LogP ~-1.15), high polarity, and zwitterionic nature at physiological pH (pKa ~2.3 for the carboxylic acid and ~8.7 for the primary amine) limit its passive diffusion across the lipid-rich sublingual mucosa [13-18]. Despite these theoretical barriers, we observed a clinically meaningful response, which may be attributed to several factors. The use of a high local concentration and prolonged mucosal contact time under the tongue facilitated partial absorption across the sublingual membrane [15]. Moreover, even small increases in plasma levodopa concentrations can result in substantial symptomatic relief in patients with PD, particularly during “off” periods [12,19]. Crushing the levodopa/carbidopa tablet into a fine powder increased the exposed surface area, promoting mucosal contact and absorption [12,19]. In addition, some of the administered dose may have been inadvertently swallowed, leading to delayed gastrointestinal absorption and contributing to the observed therapeutic effect. Overall, this suggests that while sublingual administration of levodopa/carbidopa may be pharmacologically suboptimal, it may still produce a modest but meaningful clinical effect under certain conditions, particularly when other routes or formulations are unavailable or inaccessible.

There are currently limited published case reports and clinical studies looking at crushed levodopa/carbidopa given sublingually as a potential therapeutic strategy for managing PD in adult patients in hospitals. There is only one case series (n = 2) that describes sublingual levodopa/carbidopa being used to prevent PD in perioperative patients with oropharyngeal dysphagia [10]. In this report, two elderly male patients with PD experienced challenges with perioperative levodopa/carbidopa administration due to surgical constraints that precluded oral intake. One patient was intubated following multiple abdominal surgeries, and the other could not be given a nasogastric tube due to intraoperative hypotension during shoulder surgery. Crushed levodopa/carbidopa was successfully administered sublingually in both cases to prevent the onset of parkinsonian symptoms and maintain symptom control. Both patients recovered uneventfully and were transitioned back to oral levodopa/carbidopa postoperatively. Our novel case report contributes to this limited body of evidence, while having the limitations of being a single-patient, observational case, which lacks supporting pharmacokinetic data.

## Conclusions

This case report highlights a potential alternative route for levodopa/carbidopa administration in situations where oral intake is not feasible and other non-oral formulations (e.g., rotigotine patches, apomorphine sublingual films, levodopa intestinal gels, or rectal administration) are unavailable, inaccessible, or not acceptable. While sublingual administration may offer a practical and readily implementable option in select cases, its pharmacokinetic profile, clinical efficacy, and optimal dosing remain poorly defined. The observed benefit in our case suggests that sublingual levodopa/carbidopa could help mitigate motor fluctuations and complications associated with delayed or missed doses in hospitalized patients. However, this approach should be considered exploratory, and further research is needed to establish its reliability, absorption characteristics, and broader applicability in clinical practice.
